# Software defect prediction using learning to rank approach

**DOI:** 10.1038/s41598-023-45915-5

**Published:** 2023-11-02

**Authors:** Ali Bou Nassif, Manar Abu Talib, Mohammad Azzeh, Shaikha Alzaabi, Rawan Khanfar, Ruba Kharsa, Lefteris Angelis

**Affiliations:** 1 Department of Computer Engineering, University of Sharjah, Sharjah, United Arab Emirates; 2 Department of Computer Science, University of Sharjah, Sharjah, United Arab Emirates; 3 Department of Data Science, Princess Sumaya University for Technology, Amman, Jordan; 4 Department of Statistics and Information Systems, Aristotle University of Thessaloniki, Thessaloniki, Greece

**Keywords:** Electrical and electronic engineering, Computer science, Scientific data

## Abstract

Software defect prediction (SDP) plays a significant role in detecting the most likely defective software modules and optimizing the allocation of testing resources. In practice, though, project managers must not only identify defective modules, but also rank them in a specific order to optimize the resource allocation and minimize testing costs, especially for projects with limited budgets. This vital task can be accomplished using Learning to Rank (LTR) algorithm. This algorithm is a type of machine learning methodology that pursues two important tasks: prediction and learning. Although this algorithm is commonly used in information retrieval, it also presents high efficiency for other problems, like SDP. The LTR approach is mainly used in defect prediction to predict and rank the most likely buggy modules based on their bug count or bug density. This research paper conducts a comprehensive comparison study on the behavior of eight selected LTR models using two target variables: bug count and bug density. It also studies the effect of using imbalance learning and feature selection on the employed LTR models. The models are empirically evaluated using Fault Percentile Average. Our results show that using bug count as ranking criteria produces higher scores and more stable results across multiple experiment settings. Moreover, using imbalance learning has a positive impact for bug density, but on the other hand it leads to a negative impact for bug count. Lastly, using the feature selection does not show significant improvement for bug density, while there is no impact when bug count is used. Therefore, we conclude that using feature selection and imbalance learning with LTR does not come up with superior or significant results.

## Introduction

Recently, software systems have experienced massive growth in number, size, and complexity. These dramatic changes have elevated the demand on software testing, which is costly and time-consuming^1^. With the aim of efficient allocation of software testing resources, Software Defect Prediction (SDP) has been an active area of research. SDP is the predictive process of identifying software modules with defect- or bug-proneness based on their method-level and class-level metrics^2^. It is a helpful tool during the testing phase to improve quality, reliability, and cost reduction. Previous SDP models used classification Machine Learning (ML) algorithms, such as Support Vector Machine (SVM)^3^, Random Forest (RF)^4,5^, K-Nearest Neighbor (KNN)^6^, and Naïve Bayes (NB), to provide binary classifications for the existence of defects in software modules^7,8^. SDP as a classification tool proved its importance. Still, its outcomes were insufficient in practice, as they do not account for the importance of a defective module and which modules should be examined first^9^. To produce more accurate resource assignments, researchers started to study SDP as a ranking problem using Learning-to-Rank (LTR) or regression algorithms. Instead of finding an explicit defect count prediction, ranking algorithms work towards ordering modules according to their defects or defect densities such that, for instance, the module with the highest ranking is assigned the most testing resources^10^.

**Figure 1:**
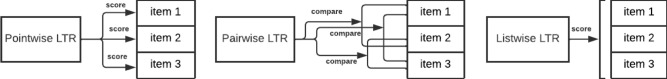
Pointwise, pairwise, and listwise LTR.

LTR is an algorithm of machine learning that builds a function to solve ranking problems on queries. It works by predicting a score in each instance, and the instances are then sorted based on the score assigned by the ranking model^11^. LTR is beneficial for many applications in information retrieval, such as e-commerce, social networks, and recommendation systems. It has proven its performance in other applications like machine translation, computational biology, recommender systems, and SDP in software engineering^12^. LTR algorithms can be classified into three approaches based on their ranking mechanism: pointwise, pairwise, and listwise, as illustrated in Fig. 1. The pointwise approach takes an individual item from the list and trains a regressor on it to predict how relevant it is for the query. The score of each item in the list (in our case, each software module) is independent of the scores of other modules. The final ranking is achieved by sorting the resultant list by the scores of the software modules^13^. The pairwise approach looks at a pair of software modules at a time. Given a pair of modules, it tries to come up with the optimal ordering for that pair and compare it to the actual ranks of these pairs of modules. The listwise approach treats the whole list as an entity and predicts the optimal ordering for each module. It uses probability models to minimize ordering errors^14^.

This research paper proposes a comprehensive comparison study of the listwise LTR approach for the SDP, starting by importing datasets that contain previous details about software modules (i.e., quality metrics and the number of bugs in each module). Subsequently, we build the SDP model by training a regression algorithm and optimizing it using Grid Search with Fault-Percentile-Average as the objective function to achieve better ranking accuracy^15^. Evaluation is the last step, yet the most essential, because it ensures the quality and reliability of the model^16^. To further analyze the proposed process and provide the desired solutions, we address the following research questions:

**RQ1.** What is the role of the target variables on the performance of the employed LTR techniques?

Two target variables are studied in this research paper: bug count and bug density. Bug count refers to the number of bugs present in a module. Bug density is a measure of how frequently a bug appears per line of code. Bug density gives a better indication of which modules require more testing resources. Given two modules with the same number of bugs, the module with a smaller number of lines of code (LOC) has a higher testing priority, as it has a higher bug density^17^.

**RQ2.** What is the average improvement when using imbalanced learning with LTR techniques?

Most SDP datasets have an imbalanced distribution with an excess of zero-count observations. Imbalanced datasets negatively affect performance, as the model is likely to be influenced by the excessive observations^18,19^. Typically, SDP datasets are imbalanced where the non-defective modules outnumber the defective modules. In this paper, we study the impact of random under-sampling of the zero-count instances (non-defective modules) on the performance of LTR techniques^20^.

**RQ3.** What is the role of feature selection in the accuracy of LTR techniques?

Feature selection is an essential preprocessing technique that can improve the execution time and accuracy of ML models, especially in SDP^21,22^. Features irrelevant to the target value can affect the overall performance of the model^23^. Feature selection is the process of choosing the most relevant attributes to train the model and enhance prediction outcomes. In this study, we apply the Information Gain (InfoGain) method to eliminate unrelated features and select the most related ones^9^.

The rest of the paper proceeds as follows. Section "Literature review" discusses the related work and relevant literature. Section "Research methodology" highlights the methodology for building and evaluating the model. Section "Results" illustrates the experimental work and the results, while Section "Threats to validity" mentions threats to validity. Lastly, Section "Conclusion" provides a summarized conclusion and suggests directions for future work.

## Literature review

SDP has been a hot topic for many years. Researchers have conducted a large number of studies, explored many areas in the field, and applied various algorithms seeking better accuracy. This section reviews the related works and algorithms used to construct SDP models; however, it focuses on the SDP ranking models as they are most relevant to our study.

### Software defect prediction

Each dataset in the classification SDP model is defined as $D=[{{\varvec{x}}}_{i},{y}_{i}]$, for modules $i \in [1,n]$. ${{\varvec{x}}}_{i}=[{x}_{i1},{x}_{i2},{x}_{i3},\dots {x}_{im}]$ represents a vector of $m$ independent features (i.e., quality metrics) of the ${i}^{th}$ module. The dependent variable is ${y}_{i}\in \{-\mathrm{1,1}\}$. “1” represents the defective modules; “-1” represents the clean ones. The equation ${y}_{i}{\prime}=f\left({{\varvec{x}}}_{i}\right)$ represents the ML classification models that predict ${y}_{i}{\prime}$ depending on the ${{\varvec{x}}}_{i}$. Different algorithms $f\left(.\right)$, provide different accuracies in classifying the modules.

ML Classifiers have been the most popular approach in the field of SDP. Guo et al.^4^ constructed a classification model using RF on five NASA datasets and used Defect Detection Rate (PD) and Overall Accuracy (ACC) to evaluate the model. Alsghaier and Akour^3^ used the Genetic Algorithm (GA) and Particle Swarm Optimization (PSO) to optimize the SVM algorithm and applied the model to 24 NASA datasets,they used accuracy, recall, precision, specificity, and F-measure as evaluation measures. As the number of used classifiers increased, researchers started conducting studies to compare them. Bansal^24^ constructed a comparative analysis of six classification algorithms. He compared the results from using static metrics, results from using change metrics, and results from combining both. Bansal evaluated the models using the Matthews Correlation Coefficient (MCC) and Area Under the Curve (AUC). He found that models trained by a combination of static and change metrics performed the best. Moreover, models that used only change metrics slightly outperformed models that used only static metrics.

Li et al.^25^ conducted a benchmark study using 17 classifiers on 27 datasets from MDP and Github. After applying AUC to evaluate the classifiers, Li et al. found that RF and Multilayer Perceptron (MLP) achieved good results,however, there was no significant difference in performance among the 17 classifiers. Weyuker et al.^26^ compared four modeling methods (NBR, RF, Recursive Partitioning (RP), and Bayesian Additive Regression Trees (BART)) and found that NBR and RF significantly surpassed RP and BART. Previous studies have found that eliminating irrelevant features using feature selection can significantly improve the model^27–29^. Chen et al.^30^ applied multi-objective optimization for feature selection. Yang et al.^31^ utilized InfoGain to select the most relevant three metrics of each dataset and found that most of the selected metrics were change metrics. Wang et al.^28^ applied a threshold-based feature selection method. They discovered that three features can construct an effective classifier and that model prediction improved when they removed 98.5% of the features.

Balogun et al.^32^ conducted a comparative study between three of the most widely used filtering approaches for dimensionality reduction (Chi-Square (CS), ReliefF (REF), and InfoGain)), for two of the SDP classification algorithms (NB and DT). They also proposed the “Rank Aggregation-Based Multi-Filter Feature Selection (RMFFS)” method, aggregating the resulting features from multiple filters. Balogun et al. found that RMFFS performed noticeably better than the solo techniques, especially the G-Mean, which resulted in the best outcomes.

Shin et al.^33^ experimented on 32 SDP datasets with LIME and Breakdown to determine whether they reasonably explain the classification results from 5 classifiers (see Table 1). Their experiments revealed that none of the mentioned methods consistently explained different settings, making them unreliable for practical use.

**Table 1:** Summary of literature review.

Title	Algorithms	Used datasets	Performance measurements	Key findings
Tong et al. “SHSE: A subspace hybrid sampling ensemble method for software defect number prediction” (2022)^40^	SHSE	“JDT, PDE, Equinox, Lucene, Mylyn, ant-1.3, ant-1.4, ant-1.5, ant-1.6, ant-1.7, camel-1.0, camel-1.2, camel-1.4, camel-1.6, ivy-1.12, ivy-2.0, jEdit-3.2.1, jEdit-4.0, jEdit-4.1, jEdit-4.2, log4j-1.0, log4j-1.1, synapse-1.0, synapse-1.1, synapse-1.2, xalan-2.4, xalan-2.6”	FPA	The FPA improved using SHSE by approximately 8–15% on the zero-inflated and resampling methods
Yu et al. Predicting the precise number of software defects: Are we there yet? (2022)^41^	“PR, ZIPR, NBR, ZINBR, HR, GP, NNR, DTR, LR, BRR, SVR, KNR.”	“Ant 1.6, Ant 1.7, Camel 1.2, Camel 1.4, Camel 1.6, Jedit 4.0, Jedit 4.1, Jedit 4.2, Poi 2.0, Poi 2.5, Poi 3.0, Xalan 2.4, Xalan 2.5, Xalan 2.6, Xalan 2.7, Xerces 1.2, Xerces 1.3, Xerces 1.4”	AAE and Pred(0.3)	Predicting the exact number of module errors is still tricky in the SDP field
Alazba and Aljamaan “Software defect prediction using stacking generalization of optimized tree-based ensembles” (2022)^49^	Grid search with ensemble classifiers “Ada, RF, ET, GB, HGB, XGB, CATS”	“JM1, KC1, PC5, Eclipse JDT Core, Eclipse PDE UI, camel 1.2, lucene 2.4, prop-1, prop-2, prop-3, prop-4, prop-43, prop-5, xalan 2.5.0, xalan 2.6.0, Eclipse 2.0 Eclipse 2.1, Eclipse 3.0, SWT, Debug”	F-measure, AUC	the RF and XGB outperformed all other tree-based classifiers
Alsghaier et al. “Software fault prediction using particle swarm algorithm with GA and support vector machine classifier” (2021)^3^	“SVM, PSO, GA.”	“CM1, KC2, KC3 KC4, MC1, MC2 MW1, PC1, PC2, PC3, PC4, PC5, ant-1.6, camel-1, ivy-1.4, ivy-2.0, jedit-4.1, log4j, lucene-2.2, poi-2.5, poi-3.0, synapse-1.2, xalan-2.5, xerces-1.3.”	accuracy, SD, error rate, specificity, precision, recall, and F-measure	Integrating GA with SVM and PSO improves the results when applied to large-scale and small-scale datasets and overcomes the limitations of previous studies
Basal et al. “Comparative analysis of classification methods for prediction software fault proneness using process metrics” (2021)^24^	“NB, Decision Tree, SVM, KNN, Logistic Regression, and RF.”	“Ant1.4, Ant1.5, Ant1.6, Ant1.7, Jedit4.0, Jedit4.1, Synapse1.1, Synapse1.2, Xalan2.5.0, Xalan2.6.0, Xalan2.7.0, Xerces1.2.0, Xerces1.3.0, Xerces1.4.4.”	AUC and MCC	Models were trained by a combination of static and change metrics and performed the best Models that used change metrics slightly outperformed models that used static metrics
Balogun et al. “Empirical analysis of rank aggregation-based multi-filter feature selection methods in software defect prediction” (2021)^32^	“NB and DT with Chi, InfoGain and ReF”	“CM1, KC1, KC2, KC3, MW1, PC1, PC3, PC4, PC5”	Accuracy, F-measure, recall, precision, AUC	The G-mean RMFFS outperformed solo methods in filtering the features and reducing the dimensionality
Shin et al. “Explainable software defect prediction: are we there yet?” (2021)^33^	LIME and Breakdown to explain the results of “Averaged Neural Network (AVNNet), Extreme Gradient Boosting Tree (xGBTree), DT, RF, and Gradient Boosting Machine (GBM)”	“ActiveMQ versions 5.0, 5.1, 5.2, 5.3 ,5.8 Camel versions 1.4, 2.9, 2.10, 2.11 Derby versions 10.2, 10.3, 10.5 Groovy versions 1.5.7, 1.6.0.b1, 1.6.0.b2 HBase versions 0.94, 0.95.0, 0.95.2 Hive versions 0.9, 0.10, 0.12 JRuby versions 1.1, 1.4, 1.5, 1.7 Lucene versions 2.3, 2.9, 3.0, 3.1 Wicket versions 1.3.b1, 1.3.b2, 1.5.3.”	Hit-rate and rank-difference	The LIME and Breakdown yielded inconsistent results for different datasets and classifiers. Thus, they are not reliable in explaining the SDP ML conclusions
López-Martín et al. “Transformed K-nearest neighborhood output distance minimization for predicting the defect density of software projects”^34^	TkDM	Four datasets selected from the ISBSG	MAR, MdAR, SA, and Effect Size	K = 6 yielded the best results when using the TkDM algorithm to predict the Defect Density (DD) based on the Function points (FP) TkDM outperformed other algorithms (e.g., SVR, NN), resulting in the least AR and highest SA
Bal and Kumar “WR-ELM: weighted regularization extreme learning machine for imbalance learning in software fault prediction” (2020)^39^	WR-ELM	“Ant 1.5, Camel 1.0, Jedit 4.3, Ivy 1.4, 2.0, Poi 2.0, Synapse 1.0, Prop 1 (V4, V40, V185), Prop 2 (V9, V44, V128, V164, V192), Prop 3 (V255, V236, V245, V265), Prop 4 (V285, V292, V305), Prop 5 (V347, V362), Prop 6 (V452, V453)	AAE, ARE, Pred(I)	The WR-ELM outperformed other techniques in handling the prediction of the minority classes in imbalanced datasets
Yang et al. “A learning-to-rank approach to software defect prediction” (2020)^31^	“Linear regression optimized with CoDE.” FPA	“Eclipse_II_File2.0, Eclipcs_II_File2.1, Eclipcs_II_File3.0, Eclipcs_II_Package2.0, Eclipse_II_Package2.1, Eclipse_II_Package3.0, eclipse, equinox, lucene, mylyn, pde”	FPA and CLC	Directly optimizing the ranking performance instead of predicting the exact number of defects in each module can improve ranking results
Li et al. “Evaluating software defect prediction performance: an updated benchmarking study” (2019)^25^	“Bagged MLP, artificial neural network, Boosted decision trees, CART, Logistic regression, Multilayer” “perceptron artificial neural network, Random forest, Ridge Regression, Linear support vector machine, SVM with radial basis kernel function, Alternating decision tree Tree Augmented Naive Bayes, J4.8, k-nearest neighbor, 8 Logistic model tree, Naive Bayes, Radial basis function neural network, Voted perceptron”	“CM1, JM1, KC1, KC3, MC1, MC2, MW1, PC1, PC2, PC3, PC4, PC5, Android-Universal-Image-Loader, BroadleafCommerce, MapDB, antlr4, ceylon-ide-eclipse, elasticsearch, hazelcast, junit, mcMMO, mct, neo4j, netty, orientdb, oryx, titan.”	AUC and H measure	RF and Neural Network Model (MLP) achieved good results There was no significant difference in the performance among the 17 classifiers
Yu et al. “An empirical study of learning to rank techniques for effort-aware defect prediction” (2019)^9^	“NB, LogR, CART, Bagging, RF, KNN, DTR, LR, BRR, NRR, SVR, KNR, GBR, SDGR, Ranking SVM, RankBoost, RankNet, LambdaRank, ListNet, AdaRank, Coordinate Ascent, LTR”	“Ant, Camel, Ivy, Jedit, Log4j, Lucene, Poi, Synapse, Xalan Xerces”	${Norm(P}_{opt})$ and FPA	BRR performed the best according to FPA BRR and LTR (by Yang et al.) performed the best when evaluated with FPA and ${Norm(P}_{opt}).$
Buchari et al. “Implementation of chaotic Gaussian PSO for optimize Learning-to-Rank software defect prediction model construction” (2018)^47^	“Linear regression optimized with Gaussian PSO.”	“Apache Lucene Eclipse, JDT Core Equinox. Framework Eclipse PDE, UI Mylyn, Eclipse_File2.0, Eclipse_File2.1, Eclipse_File3.0, Eclipse_Package2.0, Eclipse_Package2.1, Eclipse_Package2.0.”	FPA	Improved prediction accuracy in some datasets
Chen et al. “Applying feature selection to software defect prediction using multi-objective optimization” (2017)^30^	“MOFES”	“Ant-1.7, Camel-1.6, Ivy-2.0, Jedit-4.0, Lucene-2.4, Poi-3.0, Synapse-1.2, Velocity-1.6, Xalan-2.6, Xerces-1.4”	AUC	The proposed method achieved better performance with fewer features and acceptable cost
Wang et al. “How many software metrics should be selected for defect prediction?” (2011)^28^	“Multilayer Perceptron, KNN, and Logistic Regression.”	“Eclipse 2.0–10, Eclipse 2.0–5, Eclipse 2.0–3, Eclipse 2.1–5, Eclipse 2.1–4, Eclipse 2.1–2, Eclipse 3.0–10, Eclipse 3.0–5, Eclipse 3.0–3”	AUC	An effective classifier can be constructed with only three features The model prediction improved when they removed 98.5% of irrelevant features
Weyuker et al. “Comparing the effectiveness of several modeling methods for fault prediction” (2010)^26^	“NBR, RF, RP, BART.”	“Thirteen datasets from the NASA Metrics DataProgram repository”	FPA	NBR and RF significantly surpassed RP and BART
Guo et al. “Robust prediction of fault-proneness by random forests” (2004)^4^	RF	“CM1, JM1, KC1, KC2, PC1”	PD, ACC	The proposed methodology is more robust concerning noise than other models, works well with large-scale projects, and achieves higher accuracy

López-Martín et al.^34^ developed a novel algorithm to predict the Defect Density (DD) of projects based on function points (FP). The algorithm utilized transformation and reduction concepts to enhance and surpass the limitations of the original KNN algorithm in regression. The “transformed k-nearest neighborhood output distance minimization” algorithm (TkDM), minimizes the distance between the most similar k projects to the project whose DD is being predicted,then, it applies an inverse transformation to the output. Four datasets were selected from the ISBSG release 2018^35^ containing various projects with various development types and programming languages. López-Martín et al.^34^ chose the Mean Absolute Residuals (MAR) and Median Absolute Residuals (MdAR) as the main accuracy metrics for the evaluation of their algorithm, as well as Standardized Accuracy (SA) and effect size for further assessment of the algorithm performance. Also, they tested the algorithm against the SVR and NN models. Moreover, they tested different values for the number of neighbors (K) to choose the best one. Finally, they demonstrated that their algorithm yielded the highest SA and the least MAR and MdAR values compared to other algorithms.

### Learning to rank for software defects prediction

Recently, more research has been done on Software Number Prediction (SNP), where researchers predict the exact number of defects in the software module using regression algorithms^36–38^. Bal and Kumar^39^ studied the efficiency of the “extreme learning machine” (ELM) for imbalanced learning in SNP. They also derived a new method called “weighted regularization ELM” and evaluated it on 26 datasets using the Average Absolute Error (AAE), the Average Relative Error (ARE), and the Pred(I). Bal and Kumar^39^ found that the WR-ELM outperformed other techniques for predicting the minority classes in imbalanced datasets.

Tong et al.^40^ utilized the “subspace hybrid sampling ensemble” (SHSE) method for SNP. They evaluated their model on 27 open-source, public datasets detailed in Table 1. The work of Tong et al.^40^ resulted in an approximate FPA improvement of 8–15% compared to the previous ensemble and zero-inflated methods. A recent study by Yu et al.^41^ demonstrated that the prediction of the exact number of bugs in the software modules (i.e., SNP) is still difficult. They reached this conclusion after a detailed study using various regression algorithms, datasets, and optimization methods (see Table 1 for details). They evaluated the regression algorithms on the Average Absolute Error (AAE) and pred (0.3)^42^. Yu et al.^41^ suggested that the ranking SDP is the best approach for the regression algorithms.

In the ranking SDP model, each module in the dataset used is represented as ${{\varvec{M}}}_{i}=[{{\varvec{x}}}_{i},{y}_{i}]$, where ${{\varvec{x}}}_{i}=[{x}_{i1},{x}_{i2},{x}_{i3},\dots {x}_{im}]$ represents a vector of $m$ independent features (i.e., quality metrics) of the ${i}^{th}$ module. The dependent variable ${y}_{i} \in R$ represents the number of bugs in the ${i}^{th}$ module, or the bug density (i.e., $\frac{\#bugs}{LOC}$). $D=\{{{\varvec{M}}}_{i}=\left[{{\varvec{x}}}_{i},{y}_{i}\right]{\}}_{i=1}^{n}$ defines the software defect dataset, where $n$ is the number of modules in $D$. The goal of the LTR algorithms is to build a prediction model that ranks new modules based on the number of bugs or bug density, where ${M}_{j}>{M}_{k}$ means that module $j$ is more defect-prone than module $k$^31^.

Unlike SDP for classification, SDP for ranking is still relatively new, with fewer studies and research. Ostrand et al.^43^ performed a simple Negative Binomial Regression (NBR) on one static metric (i.e., LOC) to predict the number of defects in each module. Then, they ranked the modules according to their defect density. They evaluated the model by calculating the percentage of faults in the top 20% of modules. This produced better results than a simple regression model. Yang et al.^31^ proposed an LTR approach that optimized the linear regression model using CoDE with FPA as the objective function and the performance measurement. They demonstrated the effectiveness of the LTR approach by directly optimizing the algorithm. Yu et al.^9^ applied 23 LTR algorithms to 41 datasets from the PROMISE repository^44^, then performed ${Norm(P}_{opt})$ and FPA to evaluate and compare the algorithms. They found that Bayesian Ridge Regression (BRR) performed the best according to FPA, while BRR and LTR (by Yang et al.) performed the best when evaluated with FPA and ${Norm(P}_{opt}).$ Yu et al.^9^ divided the 23 algorithms into four categories: Classification-based pointwise approach, Regression-based pointwise approach, pairwise approach, and Listwise approach.

Some ML algorithms do not perform well with their default hyper-parameter settings. Selecting the best hyper-parameter of these algorithms can boost their predictive performance^45^. Researchers have utilized many optimization techniques to enhance and improve their model’s performance by tuning the hyper-parameters of the algorithm to minimize or maximize an objective function. Tantithamthavorn et al.^46^ applied an automated parameter optimization technique called Caret to optimize the SDP and found that the AUC improved by 0.4 points after applying Caret. Yang et al.^31^ performed CoDE optimization, with FPA as the objective function to directly optimize the ranking performance of the SDP. Canfora et al. applied GA to optimize the algorithm. Buchari et al.^47^ used a meta-heuristic chaotic Gaussian PSO for optimizing their regression model and chose FPA as their objective function. PSO was introduced by Kennedy and Eberhart^48^. They derived the algorithm from the behavior of birds and fish when they search for food in groups: every group member benefit from the knowledge of its swarm. A flock of birds can integrate the experiences of all members to find food in much less time. PSO is a heuristic algorithm used to search for the optimal maximum or minimum solution to a problem. Although PSO does not guarantee finding the real global optimal, it finds a value that is close enough to be sufficient in most cases. Alazba and Aljamaan^49^ combined ensemble learning with optimization methods. They used a grid search to find the best hyperparameters of tree-based ensemble algorithms. After assessing their approach on 21 datasets, Alazba and Aljamaan^49^ found that the RF and XGB outperformed all tree-based classifiers. Ni et al.^38^ investigated the usefulness of effort for cross-project defect prediction. The results obtained are promising and show superior results than traditional cross-project techniques.

It is important to note that some researchers proposed using the concept of effort-aware to prioritize software modules and aim to detect more bugs while inspecting a specific number of modules. For instance, Mende et al.^50^ introduced the concept of "effort-aware" and presented two strategies for evaluating EADP models. Kamei et al.^51^ found that process metrics yielded better results than product metrics in EADP models. In their work, Kamei et al.^52^ proposed an Effort-Aware Linear Regression (EALR) model, demonstrating its ability to detect 35% of defective code changes by examining only 20% of all changes. Yang et al.^53^ confirmed the effectiveness of slice-based cohesion metrics for EADP. Bennin et al.^54^ investigated optimal EADP algorithms and explored the practical benefits of data resampling techniques. Yang et al.^58^ discovered that the unsupervised method ManualUp^34^ generally outperformed several simple supervised models for change-level EADP. Fu et al.^55^ introduced the OneWay method, which utilizes the training dataset to automatically select the best software feature for ManualUp. Different studies explored various Effort Aware Defect Predictions^9,56–58^.

Qu et al.^59^ suggested integrating developer information into EADP to enhance performance. Carka et al.^60^ proposed using the normalized PofB to assess EADP performance, which ranked software modules based on predicted defect densities. Huang et al.^61^ presented the Classify Before Sorting (CBS +) algorithm for EADP, which outperformed other algorithms to identify defective changes. Compared to ManualUp, The CBS + identified a similar number of defective changes but required inspection of fewer changes and significantly reduced the Initial False Alarms. Finally, Li et al.^62^ investigated the impacts of different feature selection algorithms for effort-aware defect predictions. Finally, Multiple authors investigated the importance of effort aware methods for just in time software defect prediction^36,37,63^

## Research methodology

**Figure 2:**
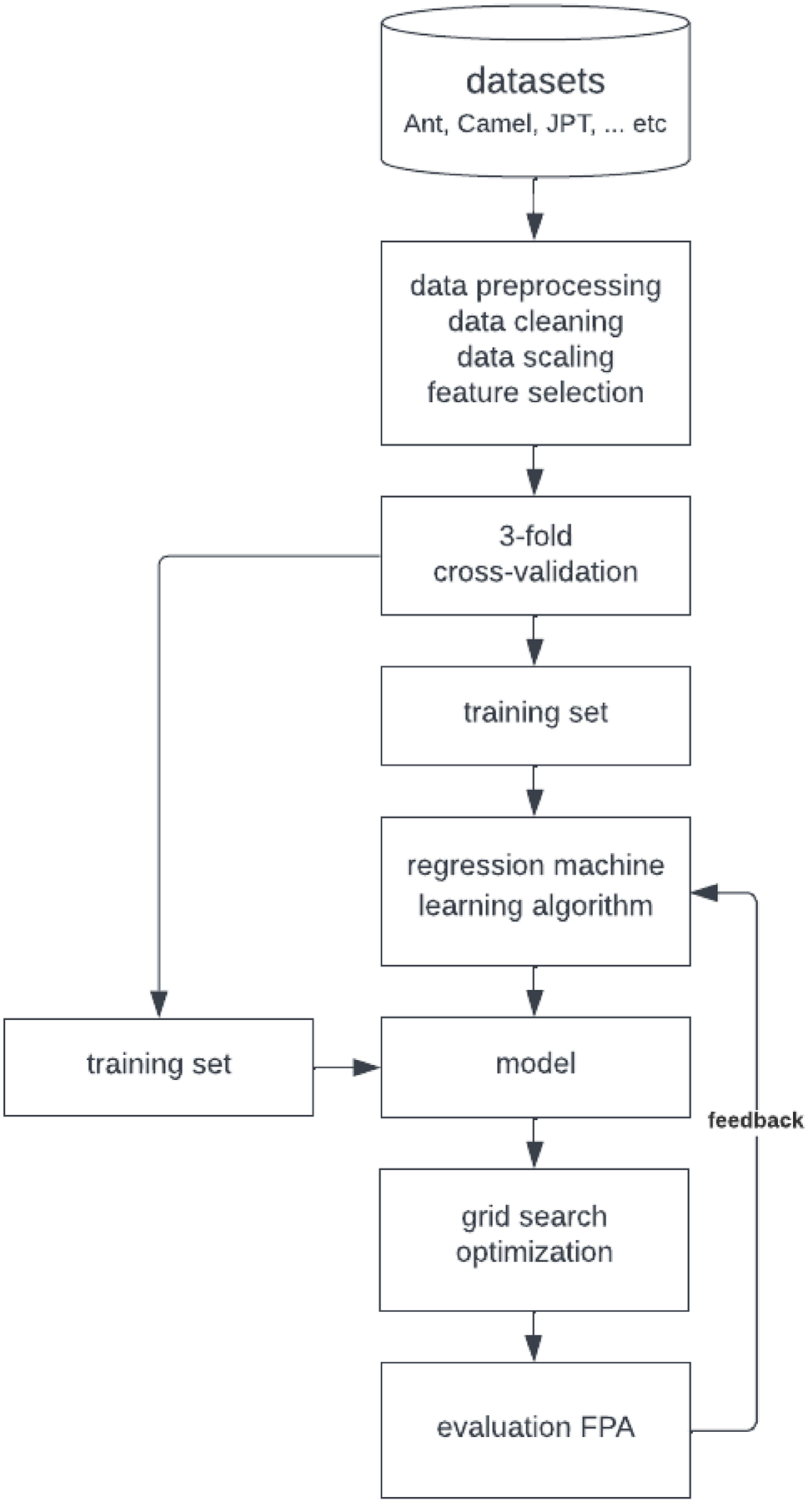
The process of building the SDP model using the LTR approach.

This section discusses the research approach for constructing different SDP ranking models. It states the characteristics of the used datasets, explains the data preprocessing and optimization techniques, explores multiple algorithms for building the regression model, and presents an evaluation strategy to assess and compare models based on various criteria. Figure 2 summarizes the conducted research methodology in this paper.

As depicted in Fig. 2, we start with an unprocessed dataset, which is imbalanced, unnormalized, and contains many inessential features. Working with raw data is always ineffective; therefore, we preprocess the data using suitable preprocessing techniques (i.e., removing outliers, data normalization, and feature selection). We then build our regression models using the best-known regression algorithms. Our experiments are done on eight algorithms: MLP, SVR, KNR, BRR, RF, XGB, ZIPR, and ZIGPR. We chose the best hyperparameters of the algorithms (except for the zero-inflated ones) using a grid search that explores many possible variants of the hyperparameters and chooses the best combination that optimizes a quality metric. In this case, we search for the hyperparameters that minimize the error of the algorithm predictions. Our approach utilizes three-fold cross-validation for fair and precise assessment and evaluation of our models. The process is performed with two target variables: bug count and bug density. Finally, we present a comprehensive comparison study between the correctness and performance of the eight models on the target variables. The rest of this section gives more details about the methodology, datasets, and metrics we adopted.

### Datasets

Most previous studies in this field use datasets from the BUG PREDICTION and PROMISE repositories^44,64^. These datasets belong to public projects and contain different types of quality metrics. Early datasets contain method-level metrics (e.g., LOC, McCabe Complexity, and Halstead metrics). However, more recent datasets employ object-oriented and change metrics^65^. Tables 2 and 3 show static and change metrics from Bansal research^24^.

**Table 2:** Static code metrics description^24^.

Static metrics	Description
WMC	Weighted method per class
DIT	Depth of inheritance tree
NOC	Number of children
CBO	Coupling between objects
RFC	Response for a class
LCOM	Lack of cohesion in methods
LCOM3	Lack of cohesion in methods version 3
NPM	Number of public methods
DAM	Data access metric
MOA	Measure of aggregation
MFA	Measure of functional abstraction
CAM	Cohesion among methods
IC	Inheritance coupling
CBM	Coupling between methods
AMC	Average method complexity
Ca	Afferent coupling
Ce	Efferent coupling
Max (CC)	Maximum McCabe’s complexity
Avg (CC)	Average McCabe’s complexity
LOC	Line of code

**Table 3:** Change metrics description^24^.

Change metrics	Description
NR	Number of revisions
NDC	Number of distinct committers
NML	Number of modified lines
NDPV	Number of defects in previous versions

This research paper uses datasets from public projects to train and test the model. These datasets have different attributes and instances. D’Ambros et al.^64^ collected the bug prediction repository that consists of PDE and JDT datasets. On the other hand, Ant, Camel, Ivy, Jedit, Lucene Poi, Synapse, Velocity, Xalan, and Xerces are parts of the Promise Software Engineering Repository. Table 4 summarizes the characteristics of each dataset^44,64^.

**Table 4:** Dataset characteristics.

Dataset	Name	Number of attributes	Number of instances	Total number of bugs	%buggy modules
PROMISE	Ant-1.7	21	745	338	45.4
	Camel-1.0		339	14	4.1
	Camel-1.6		965	500	51.8
	Ivy-2.0		352	40	11.4
	Lucene-2.0		196	93	47.4
	Poi-2.0		314	39	12.4
	Synapse-1.0		157	21	13.4
	Synapse-1.2		257	145	56.4
	Velocity-1.6		229	190	83.0
	Xalan-2.4		723	156	21.6
	Xerces-1.2		440	115	26.1
	Xerces-1.3		453	193	42.6
Bug prediction dataset	PDE R2_0	49	576	242	42.0
	PDE R2_1		761	231	30.4
	PDE R3_0		881	584	66.3
	PDE R3_1		1108	773	69.8
	PDE R3_2		1351	1124	83.2
	JDT_R2_0		2397	1102	45.97
	JDT_R2_1		2743	876	31.94
	JDT_R3_0		3420	1320	38.60
	JDT_R3_1		3883	1272	32.76
	JDT_R3_2		2233	816	36.54

### Data preprocessing

Data preprocessing is an essential step in building ML models. The Garbage in Garbage Out principle (GIGO)^66^ highlights the importance of the data preprocessing stage in data analysis. The results depend heavily on the completeness, quality, integrity, and consistency of the data fed to the model. Therefore, increasing the data quality can considerably boost the reliability of the results. Data preprocessing techniques include data normalization, under-sampling, and feature selection^67,68^. Normalization is transforming the data in all attributes into similar ranges to avoid problems related to the considerable difference between the ranges. The dataset is normalized using the min–max normalization technique^69,70^. This technique transforms all data points into values between zero and one using (1).

1 $$\begin{array}{c}{x}_{sc}= \frac{x-{x}_{min}}{{x}_{max}-{x}_{min}}\end{array}$$

Feature selection is a principal data preprocessing technique that enhances performance and reduces complexity by removing irrelevant attributes. This research utilizes InfoGain to select the most crucial features and demonstrates that there are cases where we can achieve the same results using a small percentage of the attributes^71^. InfoGain measures the dependencies between each attribute and the target value,after that, it ranks the variables based on the gain in the target variable (i.e., bug count or density). The attributes that reduce the uncertainty of the target have higher information gain values and thus have a higher chance of being selected^67,71^. Equations (2), (3), and (4) are used to calculate the InfoGain.

2 $$H\left(Y\right)= -\sum_{y\in Y}p(y){log}_{2}p(y)$$

where $H(Y$) is the entropy of the target variable Y, and $y$ is each class in Y; however, since the entropy expects a discrete number of classes, we will convert the bug density into discrete ranges, then apply Eq. (3).

3 $$H\left(Y|X\right)= -\sum_{x\in X}p(x)\sum_{y\in Y}p(y|x){log}_{2}p(y|x)$$

The result “$H\left(Y|X\right)$” is the conditional entropy of the target variable $Y$ given a feature $X$. Lastly, Eq. (4) finds the gain in $Y$ after using the feature variable $X$.

4 $$I\left(X,Y\right)=H\left(Y\right)-H(Y|X)$$

These formulas are applied to the features (one at a time) to select the most relevant ones.

### Model selection and optimization

The comparative study utilizes five state-of-the-art supervised machine learning algorithms to construct a regression model that learns from known observations to predict the bug density and bug count of new observations. These models are: SVR, MLP, KNR, BRR, RF, and XGB. The study also uses the famous zero-inflated models (i.e., ZIPR, ZIGPR) to compare results and better understand trends and observations. SVR is a generalized linear regressor that predicts based on constructing a hyperplane with the maximum margin of the samples. MLP is a neural network model with input, output, and multiple hidden layers. The model is designed to discover complex hidden patterns in the data and can work for regression and classification. BRR is based on the Bayes theorem and supposes that the software features are independent. It dramatically simplifies the complexity of Bayesian methods. KNR predicts the number of bugs of the new software modules based on the number of bugs of the nearest one or several software modules. The choice of *k* number of nearest neighbors and aggregation method are the main factors of KNR. RF generates an ensemble model with essential decision trees. It randomly samples each instance to train different decision trees. XGB is an optimized distributed gradient boosting algorithm robust enough to handle various data types, relationships, and distributions. ZIFR and ZIGPR are regression techniques designed to count data with an excess of zero counts in case of bug counts and bug density.

Creating general ML models can produce acceptable results regardless of the discussed problem, even without using the data to tune them; however, it does not achieve the most desirable performance. Model optimization is finding the hyperparameters that minimize or maximize a scoring function for a specific task. Each model has its hyperparameters with a set of possible values^72^. This research employs the Grid Search technique to uncover the optimum values of the hyperparameters. Grid Search accepts the hyperparameter names (e.g., the learning rate in MLP or the kernel in SVM) and a vector of possible values for each. Then, the function goes through all the combinations and returns a fine-tuned model using the best combination of values. Even though Grid Search can require more resources and time than other optimization methods, it works better with the SDP problem since the datasets are not enormous and most of the model's hyperparameters are non-numeric (i.e., categorical or binary). Table 5 shows the hyperparameter configuration of each algorithm used by Grid search to find the best set of parameters.

**Table 5:** Hyperparameters configurations.

Model	Hyperparameter configurations
SVR	Kernel = {‘rbf’, ‘linear’,’sigmoid’}, c = {0, 0.5, 1}
KNR	K = {1, 3,5,7,9}
BRR	Alpha = {0.01, 0.02}, max_iteration = {100, 200, 300}
RF	Number of estimators = {50, 60, 70, 80, 90}, minimum number of leaves = {3, 5, 7}
XGB	Number of estimators = {50, 60, 70, 80, 90}
MLP	Number of hidden layers = {2, 3, 4}, number of hidden neurons = {20, 30, 40, 50}

### Model evaluation (fault percentile average)

As discussed previously in the literature, FPA is a state-of-the-art performance measurement for ranking SDP models. FPA is a metric for evaluating the performance of the built models. Consider a dataset that contains $k$ modules, ${m}_{1}, {m}_{2}, {m}_{3},\dots ,{m}_{k}$ ordered in increasing value according to predicted defects where ${m}_{k}$ contains the most predicted defects. Let ${n}_{i}$ represent the actual defects in ${m}_{i}$, and the total number of actual defects is $n={n}_{1}+ {n}_{2}+{n}_{3}+ \dots + {n}_{k}$. The sum of actual defects computed from the modules with the highest numbers of predicted defects is $\sum_{i=r}^{k}{n}_{i}$. Therefore, the proportion of actual defects in the top predicted defective $r$ modules to the total number of defects is:

5 $${P}_{r}=\frac{1}{n}\sum_{i=k-r+1}^{k}{n}_{i}$$

The FPA is the average of ${P}_{r}$

6 $$FPA=\frac{1}{k}{\sum }_{r=1}^{k}\frac{1}{n}\sum_{i=k-r+1}^{k}{n}_{i}$$

The previous equation shows that FPA is the average of the proportions of actual defects in the top $r$ predicted defective modules to the total defects. Where $r=1, 2, 3, .., k$, FPA is compatible with ranking models because it takes the order of the predicted defects into account. Better models have higher FPA values because their ranking is more accurate^26^.

Our research testing plan uses k-fold cross-validation to evaluate the ML model’s reliability, avoid biased and misleading results, and get the most accurate and fair assessment of each model's performance. This approach involves testing different portions of the datasets iteratively, which allows all data points to contribute to the testing process instead of one fixed model testing. Since the observations in each dataset are limited, this study uses three-fold cross-validation, computes the quality metric (i.e., FPA) in each of the three iterations, and then finds the mean for all FPA over the iterations to achieve stable, unbiased results. After building the models, optimizing them, and computing all FPA for the models with different percentages of attributes, the following section reflects on the results and discusses the main observations and findings.

### Compliance with ethical standards

The authors would like to convey their thanks and appreciation to the “University of Sharjah” for supporting the work through the research group – Open UAE Research and Development.

### Informed consent

This study does not involve any experiments on animals.

## Results

We present the results of our comparison study on SDP in this section. We include a detailed description of how the experiments were designed, how the results were evaluated, and a discussion of the results.

**RQ1.** What is the role of the target variables on the performance of the employed LTR techniques?

To answer this research question, the eight models were first trained to predict either bug count or bug density. The FPA scores of all results were calculated, and the average score of each model was found. The eight models were compared based on the FPA scores of the two target variables and were visualized using box plots.

Table 6 presents the mean FPA results of our models, applied to the Promise and Bug Prediction repository datasets. The FPA values are in the form of “mean ± standard deviation.” A higher mean FPA indicates that the model could rank the defective modules more accurately. A higher standard deviation shows higher dispersion in FPA scores. Hence, the model has low stability and less reliability as the model gives variant results. Therefore, maximizing the mean FPA and minimizing the standard deviation is desired.

**Table 6:** Mean FPA results of the eight models.

Target Variable	MLP	SVR	KNR	BRR	RF	XGB	ZIPR	ZIGPR
Bug count	0.749 ± 0.056	0.762 ± 0.060	0.746 ± 0.055	0.770 ± 0.048	0.672 ± 0.056	**0.776 ± 0.045**	0.333 ± 0.173	0.756 ± 0.058
Bug density	**0.63 ± 0.097**	0.573 ± 0.094	0.587 ± 0.092	0.617 ± 0.102	0.567 ± 0.070	0.613 ± 0.097	0.514 ± 0.108	0.618 ± 0.098

Bold indicates best results.

The table compares the mean of the FPA results of each model for all datasets with different target variables. The first and second rows indicate the FPA scores when the target variables are bug count and bug density, respectively, with the best performance highlighted in bold type.

The best FPA scores when the target variable is bug count are achieved by MLP, SVR, KNR, BRR, XBG, and ZIGPR, ranging between 74.6 and 77.6%. On the other hand, the best scores when the target variable is bug density are produced by MLP, BRR, XGB, and ZIGPR, with scores ranging from 61.3 to 63.0%. In addition, the bug count results are more reliable, as they have a lower standard deviation than bug density results. It can further be seen that ZIPR has a contrasting behavior compared to other models since its bug density score has a higher FPA mean and a lower standard deviation compared to bug count scores.

**Figure 3:**
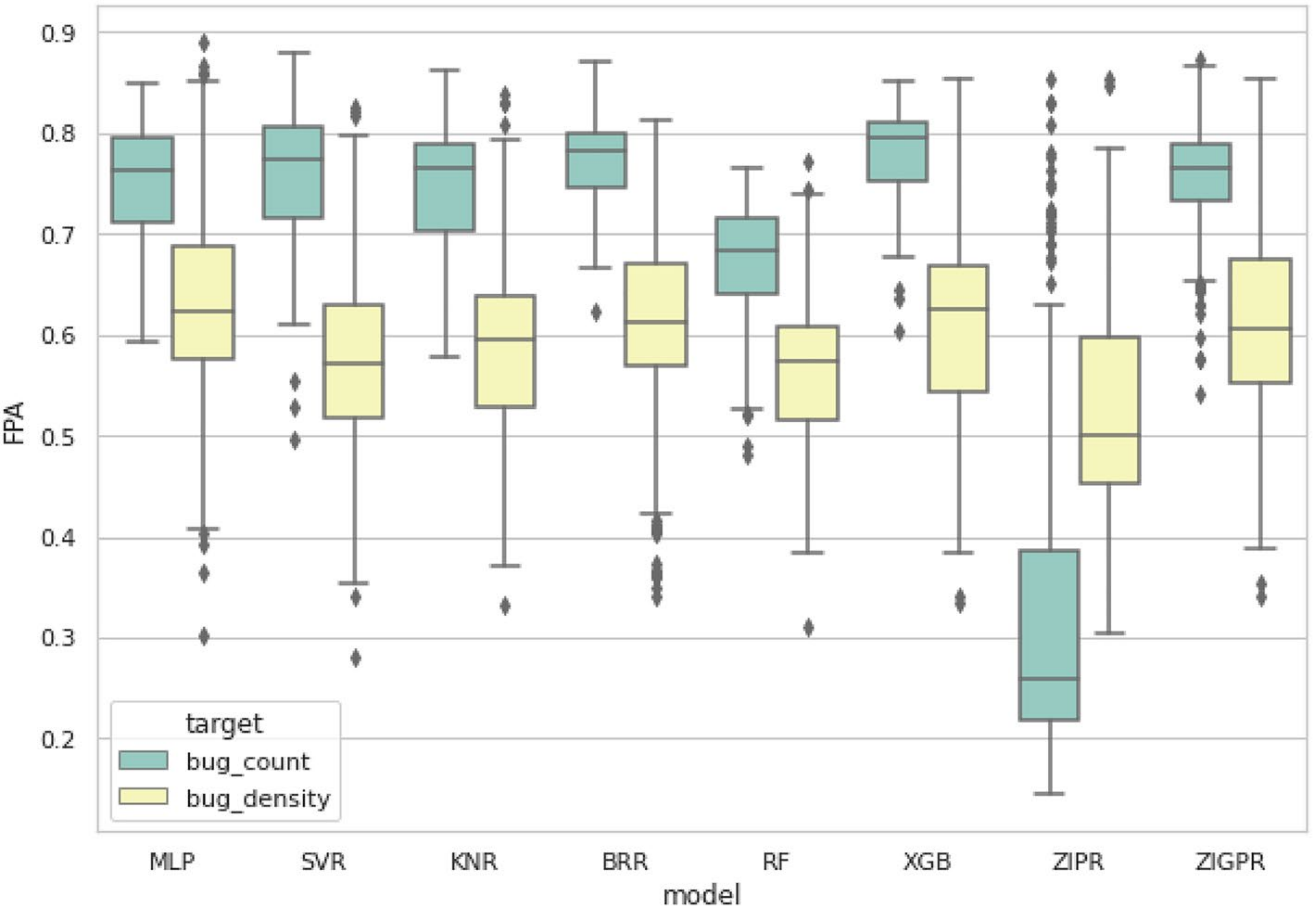
Box plot for bug count and bug density FPA results for all models.

Figure 3 visualizes the results of the table in box plots. The box plot shows the mean FPA results of the proposed models. Each model has a pair of box plots: bug count and bug density, colored in blue and yellow, respectively. The box plot shows that seven out of eight models perform significantly better when the target variable is bug count, as they have higher FPA scores and lower standard deviations since they have smaller box plots. This can be statistically proven using the non-parametric Wilcoxon test, with a 95% confidence interval applied to the bug count and bug density FPA scores. The null hypothesis states that using the bug count or the bug density as the target variable is statistically indifferent. Performing the Wilcoxon test produces a p-value of 5.706 e-09, which is less than 0.05, rejecting the null hypothesis. In contrast to the rest of the models, ZIPR produces a meager FPA score when the target variable is the bug count.

**Figure 4:**
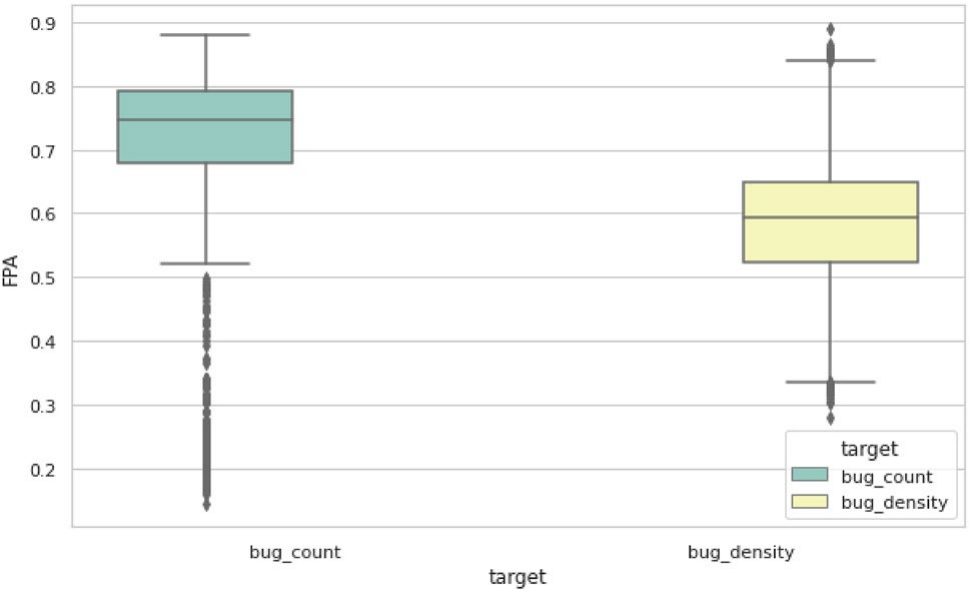
Summarized box plot for bug count and bug density FPA results.

Overall, using bug count as the target variable is more reliable and stable than the bug density, as visualized in Fig. 4. The box plot summarizes the results for all models and all datasets. The bug counts results have more outliers due to the low results of ZIPR model.

**RQ2.** What is the average improvement when using imbalanced learning with LTR techniques?

To answer this research question, the datasets were under-sampled by reducing the number of instances with a zero bug count. The under-sampling was done at different rates: 50%, 75%, 85%, 90%, and 95%, where the rate represents the percentage of non-defective samples that were randomly selected and removed from the training set. The effect of under-sampling was measured by the improvement rate calculated using (12).

7 $$improvement\,rate=1-\frac{FPA\,score\,before\,undersampling}{FPA\,score\,after\,undersampling}$$

Table 7 shows the improvement rates of results after performing under-sampling. The improvement rates are calculated relative to the results of the original dataset and are written in the form “mean ± standard deviation.” The positive improvement rate represents increasing FPA, while the negative improvement rate represents a decrease. In general, increasing the under-sampling rate slightly decreases the FPA results when the target variable is bug count, as opposed to the bug density results where the scores improved with increasing the under-sampling rate.

**Table 7:** Mean FPA results with different under-sampling rates.

Target	Under-sampling rates				
	0.5	0.75	0.85	0.9	0.95
Bug count	− 0.013 ± 0.079	− 0.041 ± 0.112	− 0.069 ± 0.139	− 0.088 ± 0.156	− 0.116 ± 0.176
Bug density	− 0.0113 ± 0.182	0.008 ± 0.184	0.024 ± 0.193	0.052 ± 0.185	0.097 ± 0.176

**Figure 5:**
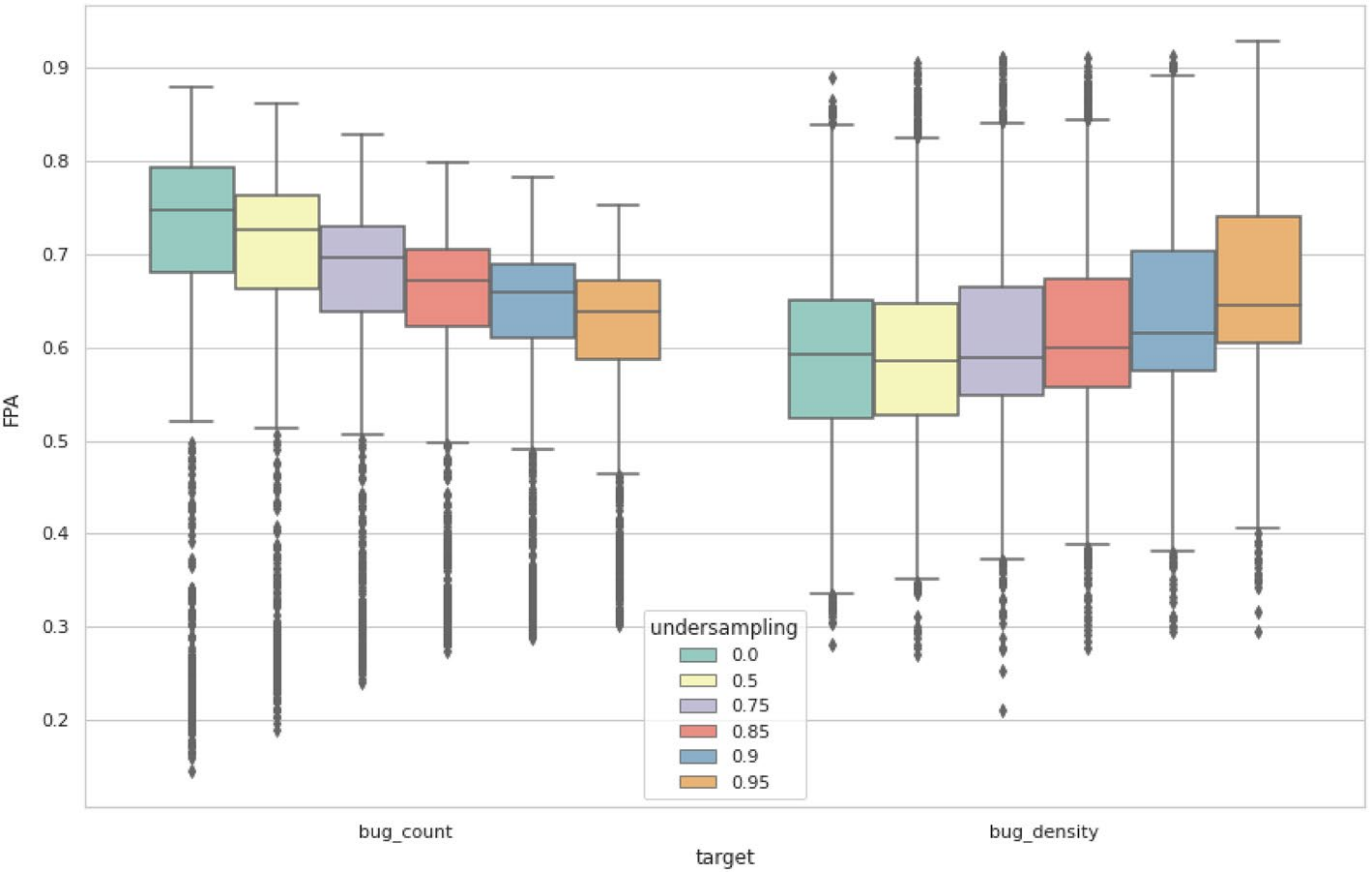
Box plot of FPA results after under-sampling.

Figure 5 illustrates the change in FPA results with the change of the under-sampling rate for bug count and bug density targets. The under-sampling rates are distinguished with different colors, as indicated in the legend of the graph. The box plot visually describes the effect of changing the under-sampling rates, as in Table 7. While under-sampling improved the results of bug density, bug count results remained higher in all cases.

**RQ3.** What is the role of feature selection in the accuracy of LTR techniques?

To answer this research question, InfoGain feature selection was first applied to the features, which ranked them based on their significance on the prediction. The models were trained using different subsets of the features, where the subset is a percentage of the top features. All results' FPA scores were calculated, and each percentage's average score was found and compared for both bug count and bug density.

Table 8 shows the results for bug count and bug density with different feature selection percentages from 10 to 100% with an increment of 10%, where 100% means all features are selected. The results are in the form “mean ± standard deviation,” with the highest highlighted in bold type. For bug count, the maximum score was achieved using 10% of the features. The maximum score was achieved for bug density using 100% or 90% of the features.

**Table 8:** Mean FPA results with feature selection.

Target	Feature selection									
	0.1	0.2	0.3	0.4	0.5	0.6	0.7	0.8	0.9	1
Bug count	**0.728 ± 0.088**	0.705 ± 0.143	0.693 ± 0.153	0.689 ± 0.171	0.688 ± 0.172	0.688 ± 0.174	0.691 ± 0.175	0.691 ± 0.173	0.691 ± 0.172	0.691 ± 0.170
Bug density	0.563 ± 0.100	0.569 ± 0.103	0.576 ± 0.099	0.585 ± 0.100	0.582 ± 0.098	0.591 ± 0.101	0.601 ± 0.102	0.608 ± 0.097	**0.611 ± 0.103**	**0.611 ± 0.101**

Bold indicates best results.

**Figure 6:**
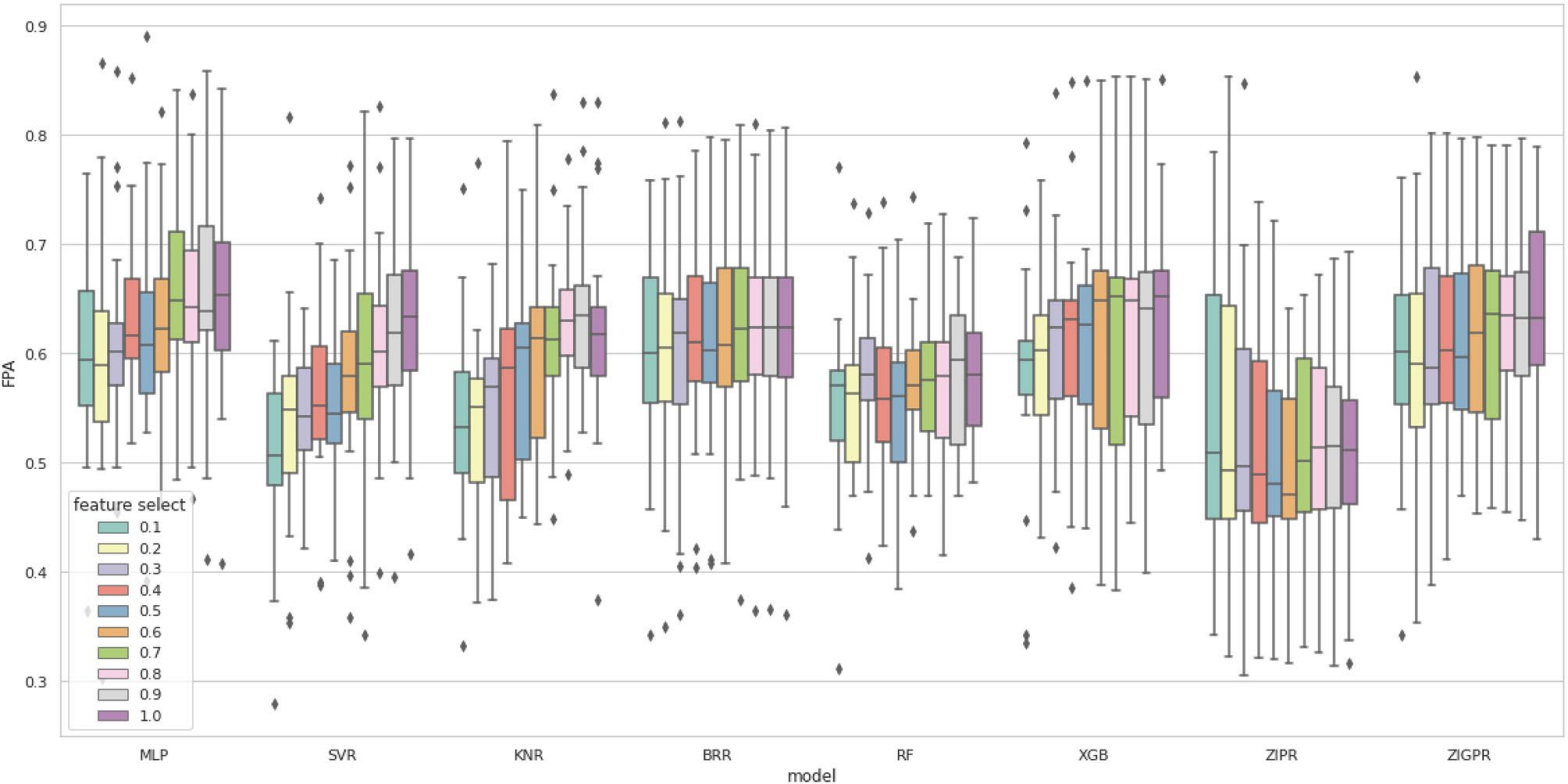
Box plot of bug count results with feature selection.

**Figure 7:**
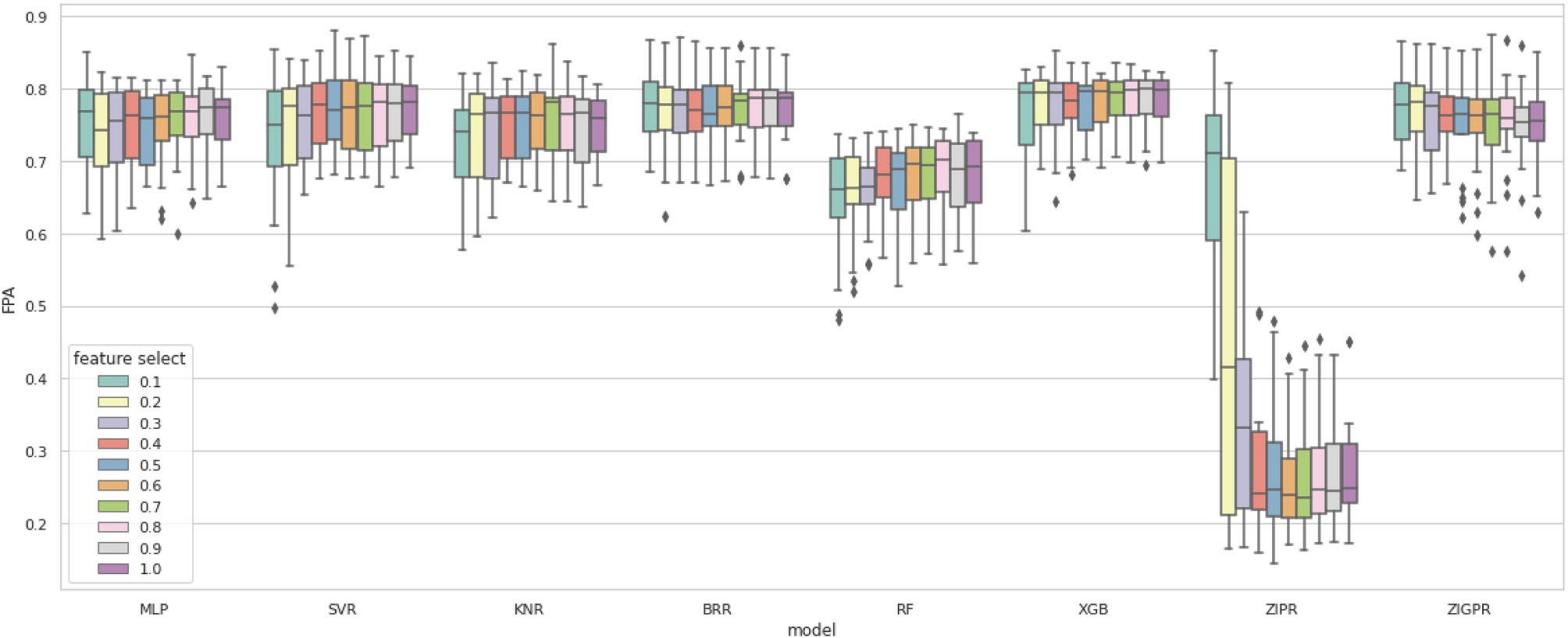
Box plot of bug density results with feature selection.

Figures 6 and 7 visualize the impact of feature selection on different models for both bug count and bug density, respectively. Figure 6 shows that most models maintained similar scores and were not significantly affected by feature selection. However, ZIPR showed unusual behavior, with shallow scores from 30 to 100% of the features but increasing sharply at 20% and 10% of the features. This shows that ZIPR is highly sensitive to the features used in the training set. In Fig. 7, most models show a decreasing FPA score as the feature selection rate decreases. Some models, such as BRR, ZIPR, and RF, show less sensitivity to features than others, such as SVR, XGB, and KNR.

**Figure 8:**
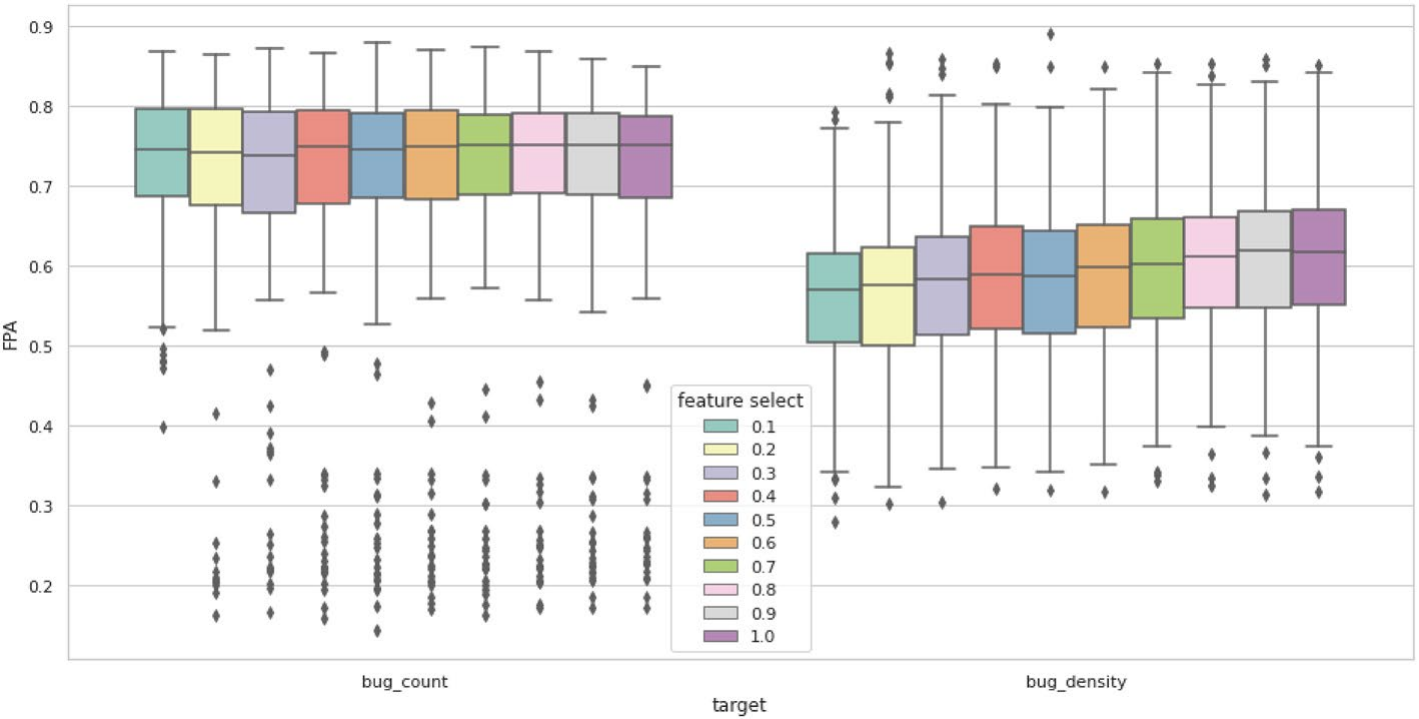
Overall box plot of results after feature selection.

Figure 8 shows the average performance of the eight models and compares the effect of feature selection on both bug count and bug density. Overall, bug count results seem to maintain the same score with all feature selection rates. This means that using the minimum number of features (10%) yields the same performance that using 100% of the features yields, reducing computational power and time requirements significantly. In contrast, bug density results showed that even the less significant features positively contribute to the model results. This was proven using the Wilcoxon test with a 95% confidence interval. The null hypothesis states that using 10% and 100% of features are statistically indifferent. Applying the test to bug count and bug density results in p-values of 0.8986 and 1.314e−10, respectively. The bug count results are statistically indifferent since the p-value is greater than 0.05. However, the p-value for the bug density results is less than 0.05; therefore, they are statistically different.

## Threats to validity

This section presents the threats that were the main factors in the validity of our comparison study. We begin with the internal validity, which is associated with the trustworthiness of the results of our study. First, data sampling methods may have affected the results, as threefold cross-validation was used. Although other sampling methods are less biased, such as tenfold and leave-one-out cross-validation, they are computationally expensive for large datasets. We tested our study on 24 datasets with large numbers of attributes and instances; therefore, using threefold cross-validation was a compromise solution. Second, machine learning models are primarily affected by the data, which is why the models used in our study were chosen based on features of our datasets, such as the distribution of the data and the characteristics of the dependent variable. While many popular performance metrics are commonly used for regression problems, such as the mean absolute error, mean squared error, and R-squared, the most appropriate metric for ranking problems is FPA. Lastly, external validity is the ability to generalize the results of the study for all datasets. This paper used 24 datasets from PROMISE and bug prediction repositories to generalize our results. We followed the approach of within-project prediction, and we did not validate the cross-project or cross-company approaches.

## Conclusion

Software Defect Prediction (SDP) is essential to software testing and quality assurance. It has become even more fundamental in recent years, as the number of programs and software products has also increased in size and complexity. In practice, project managers are not only interested in identifying defective modules but also want to rank the potential defective modules to optimize resource allocation and minimize testing costs. This is notably observed for projects with limited budgets. Thus, this paper compared multiple LTR models using two standard output metrics: bug count and bug density as target variables. It also studied the effect of using imbalance learning and feature selection on eight models with Grid Search optimization. The FPA results of the models showed that using bug count as the target variable produced higher scores and more stable results. The use of imbalance learning has shown significant improvement in the FPA scores of the bug density results but less significant on the bug count results. Finally, using feature selection with LTR has reduced the FPA score of the bug density metric while it had no impact on bug count results. Thus, we conclude that using feature selection and imbalance learning with LTR does not come up with superior or significant results. Our study has several implications for the software industry. LTR helps by ranking modules based on the defect severity, which helps to direct focus and resources to the modules that need more testing.

## Data Availability

All datasets used in this research are publicly available through PROMISE^44^, and Bug Prediction datasets^44,64^. Please check http://promise.site.uottawa.ca/SERepository/datasets-page.html and https://bug.inf.usi.ch/index.php.

## Abbreviations

SDP: Software defect prediction
ML: Machine learning
LTR: Learning to rank
FPA: Fault percentile average
MLP: Multilayer perceptron
SVR: Support vector regression
KNR: K-Neighbors regression
BRR: Bayesian ridge regression
RF: Random forest
XGB: XGBoost (extreme gradient boosting)
ZIPR: Zero inflated poisson regression
ZIGPR: Zero inflated generalized poisson regression
